# Cardiovascular risk profiling among South Asian adults in Hong Kong: a latent class analysis

**DOI:** 10.1186/s12939-025-02376-8

**Published:** 2025-01-17

**Authors:** Gary Ka-Ki Chung, Woohyung Lee, Danna Camille Vargas, Bulbul Sharma, Kai Sing Sun, Heidi Hung, Lee Sha Tong, Tsz Lui Tang, Hasiba Munir, Chi Yui Wong, Soniya Pun, Man Hin Chio, Eliza Lai-Yi Wong, Dong Dong, Eng-Kiong Yeoh

**Affiliations:** 1 JC School of Public Health and Primary Care, Faculty of Medicine, The Chinese University of Hong Kong, Hong Kong, China; 2 CUHK Institute of Health Equity, The Chinese University of Hong Kong, Hong Kong, China

**Keywords:** South Asian, Cardiovascular risk, Obesity, Latent class analysis, Demographic, Socioeconomic, Community screening

## Abstract

**Background:**

South Asians living in urbanized settings are facing disproportionate cardiovascular burden largely attributable to modifiable risk factors. Given the rapid surge in South Asian population in Hong Kong, this study aims to identify and distinguish clusters of cardiovascular risk profiles among community-dwelling South Asian adults.

**Methods:**

Between June 2022 and December 2023, 1181 South Asian adults were recruited through territory-wide outreach health assessments on lifestyle, psychological distress, obesity, clinical cardiovascular conditions, and sociodemographic factors. Latent class analysis was performed to classify the cardiovascular profiles, followed by multinomial logistic regression to identify the sociodemographic characteristics across classes.

**Results:**

Five latent classes were identified: low risk (19.6%), lifestyle risk plus distress (8.9%), obesity risk (33.4%), lifestyle risk plus distress with obesity (26.6%), and high clinical risk (11.4%). Apart from the higher clinical risk in older adults, women tended to cluster into classes with obesity, while men and the economically active were more likely in classes with poorer lifestyles and stress. Pakistani and Nepalese consistently exhibited higher risks compared to Indians, whereas the less educated tended to cluster into the high clinical risk class.

**Conclusion:**

This study revealed distinct cardiovascular risk patterns and sociodemographic features within the South Asian community in Hong Kong. The heavy burden on obesity especially in women, lifestyle and psychological risks especially in men, and low overall physical activity level may be translated into a tremendous cardiovascular disease burden in the forthcoming decades, in particular among Pakistani and Nepalese as well as the socioeconomically disadvantaged.

**Supplementary Information:**

The online version contains supplementary material available at 10.1186/s12939-025-02376-8.

## Lay summary

Our study extends the knowledge on cardiovascular risk profiling among South Asians residing in an urbanized Asian setting, and underscores significant sociodemographic disparities in their cardiovascular risks which are essential for public health stakeholders aiming at targeted preventive measures and interventions in the community.

## Key findings

- Based on data from 1181 community-dwelling South Asian adults in Hong Kong, our latent class analysis showed that less than 20% of sampled participants were classified as the “low-risk” group, implying a significant proportion at least with one domain of cardiovascular risks. Obesity, whether coupled with lifestyle and pre-existing clinical conditions, was a major concern for this fast-growing but vulnerable community in Hong Kong.
- The identified sociodemographic disparities across classes of cardiovascular risk, mainly by ethnicity, gender, and socioeconomic position, within the South Asian community in Hong Kong reiterated the need to dismiss the prevailing stereotype and community perception that assumes all South Asians are a homogeneous group for health promotion.

## Introduction

Abundant research shed light on the rising and disproportionate cardiovascular disease burden in South Asian populations, especially for South Asian diaspora living in urbanized settings [1]. As concluded by a recent scientific statement from the American Heart Association in 2018 [2], studies in the United Kingdom and the United States consistently revealed higher incidence, earlier onset, and greater standardized and proportional mortality rates from cardiovascular diseases among the South Asian community than the non-Hispanic whites and other Asian ethnic groups. Possible explanations include the predisposed genetic susceptibility to adiposity in South Asians [3], the mismatch between early and later life environments in migrant cohorts [4], as well as changes in lifestyle behaviours due to acculturation [5]. In reflection of these observations, recent guidelines on cardiovascular disease prevention included the South Asian ethnicity as a risk enhancer for cardiovascular risk prediction and treatment prescription [6, 7].

Despite the growing body of research on ethnic disparities in cardiovascular risks in high-income Western settings, evidence remains limited in developed Asian regions, in particular Hong Kong which has seen a 55.6% increase in South Asian residents from 65,521 to 101,969 in the past decade [8]. To the best of our knowledge, there is no community-based study on cardiovascular risk for the South Asian community in Hong Kong. Instead, earlier studies in Hong Kong compared the cardiovascular risks between Chinese and South Asian groups based on the medical records of patients with hypertension and diabetes in a government general out-patients clinic [9, 10]. Results indicated that patients of Nepalese, Filipino, Indian, and Pakistani descent had poorer blood pressure control, glycemic control, lipid profiles, and were more obese despite being younger than the local Chinese patients, forecasting a possible surge of cardiovascular disease burden among the South Asian community.

While further studies are warranted to examine how genetic and epigenetic factors influence the cardiovascular risks among South Asians, existing evidence suggests that a substantial portion of the elevated risk may be linked to the increased prevalence of various known modifiable risk factors [2]. Apart from the considerable burden of obesity and elevated risks of other clinical conditions including dyslipidemia, hypertension, and type 2 diabetes, exposure to unhealthy lifestyle behaviours [11] (e.g., diet, physical activity, smoking) and psychosocial distress [12] may also contribute to the excess cardiovascular risks among the South Asian community. As these domains of risk factors tend to cluster into different patterns which would in turn determine the future cardiovascular burden [13], community-based health screenings are crucial for cardiovascular risk profiling among the South Asian community in Hong Kong. Moreover, the complex interplay among ethnicity, gender, and socioeconomic position has long been recognised in shaping cardiovascular health [14]. Understanding and addressing the intersectional nature of cardiovascular risks is essential for developing effective interventions to mitigate the ethnic health inequalities. In light of the above, the present study aims to identify clusters of cardiovascular risk profiles among community-dwelling South Asian adults in Hong Kong, and to assess the differences in sociodemographic characteristics across the identified classes to inform targeted interventions.

## Methods

### Study population

Eligible participants were ethnic South Asians (i.e., Pakistani, Nepalese, Indian, Bangladeshi, and Sri Lankan), aged 18 or above, and residing in Hong Kong. In total, 1181 participants were recruited between June 2022 and December 2023 through territory-wide community outreach, carried out by all publicly-funded ethnic minority social services outreaching teams, covering all 18 administrative districts of Hong Kong. To maximize the reach of the recruitment, different channels were used by the outreaching teams, including in-person approach, telephone, and digital means considered most effective based on expert opinion and the experience of project team, for reaching South Asian community in Hong Kong. All participants signed a standardized consent form, and those who were unable to give consent were excluded [15].

### Measurement

#### Interviewer-administered survey

Surveys were administered by trained researchers, with South Asian interpretation provided as necessary, by experienced community health workers native in the relevant language. Demographic information on age, gender, marital status, household size, ethnicity, and length of stay in Hong Kong (i.e., < 4 years, 4–7 years, > 7 years (i.e., the threshold for becoming a permanent resident), and being born in Hong Kong) was collected. As for socioeconomic factors, education level was classified into ‘primary and below’, ‘secondary’, and ‘post-secondary’. For economic activity status, participants were grouped into ‘employed/self-employed’ or ‘economically inactive’ (i.e., unemployed, retired, and students). Participants were also asked about their Comprehensive Social Security Assistance (CSSA) status to determine whether they receive the means-tested financial assistance by the Hong Kong government.

In addition, lifestyle behaviours were assessed with reference to the Simple Lifestyle Indicator Questionnaire (SLIQ) [16]. Frequencies of consumption of leafy green vegetables and fruits were estimated by two separate questions, each with options ranging from ‘less than 1 time/week’ to ‘2 or more times/day’. Participants reporting less than 1 time/day were considered as low intakes of vegetable and fruit, respectively. Physical activity was assessed by three items on the frequency of light, moderate, and vigorous exercise for at least 30 min at a time per week, and then categorized into ‘none or light exercise only’ and ‘at least some moderate or vigorous exercise’. Smoking status was classified as ‘ever-smokers’ and ‘never-smokers’. Stress was also assessed using a reversely coded 6-point Likert scale with higher scores indicating greater stress. Participants reporting a score of 4 or above (i.e., upper half of the 6-point scale) were considered having psychological distress. In addition, depressive symptoms were screened using the Patient Health Questionnaire-2 [17]. Participants with a score of 3 or greater indicated presence of depressive symptoms.

#### Anthropometric assessment

Height, weight, and waist circumference were measured by trained community health workers. Participants were required to take off their shoes and heavy clothing and were asked to stand with their arms relaxed in a neutral position with their feet together. Portable digital scales (Omron HBF-514C) were used to measure participant’s weight in kilograms and body fat percentages to one decimal place. Portable stadiometers were used to measure height in centimetre to one decimal place. For waist circumference measurement, participants were asked to stand straight and breath normally while relaxing the abdomen. Circular tapes, in centimetre to one decimal place, were tightly compressed over participant’s clothing. An ethnic specific BMI cut-off of ≥ 27.5 kg/m^2^ was adopted for general obesity to account for the higher risk of type 2 diabetes among South Asian populations compared to other ethnic groups [18]. As previous research found that BMI is a weak risk factors of cardiovascular mortality [19], alternative obesity measures were also used to measure abdominal obesity defined as waist-to-height (WHtR) ratio above 50% [20] and high body fat percentage based on age-gender specific thresholds proposed in previous research for Asian populations [21].

#### Biomedical measurement

Biomedical measurements of clinical conditions were performed by registered nurses during community health outreach. Blood pressure was measured on both arms simultaneously for three times at one-minute intervals using the Watch BP office AFIB device. The average of three readings on the arm with the higher blood pressure value was used. Hypertension was defined as average systolic blood pressure ≥ 140 mmHg, and/or average diastolic blood pressure ≥ 90 mmHg, and/or self-reported physician-diagnosed hypertension [22]. Blood glucose was measured by capillary blood sample using Rightest GM700SB blood glucose monitor. Diabetes mellitus was defined as fasting plasma glucose ≥ 7 mmol/L, and/or random blood plasma glucose ≥ 11.1 mmol/L, and/or self-reported physician-diagnosed diabetes mellitus [23]. Blood cholesterol was measured by capillary blood sample using the Mission Ultra Cholesterol Monitoring System. Hypercholesterolemia was defined as plasma cholesterol ≥ 6.2 mmol/L and/or self-reported physician-diagnosed high cholesterol [24].

### Statistical analysis

Descriptive statistics of the sample characteristics are presented as mean with standard deviation (SD) for continuous variables and frequencies with percentages for categorical variables. Latent class analysis was performed to classify the cardiovascular profiles based on the above-mentioned lifestyle behaviours (4 indicators), psychological symptoms (2 indicators), obesity measures (3 indicators), and clinical conditions (3 indicators), starting from the 1-class model to the 8-class model. Multiple information criteria, including Akaike’s information criterion (AIC), Bayesian information criterion (BIC), consistent Akaike’s information criterion (CAIC), and sample-size-adjusted Bayesian information criterion (ssBIC), were used to estimate model fit. Lower values of information criteria indicate better model fit. The Vuong-Lo-Mendell-Rubin likelihood ratio test (VLMR LRT) was employed to test whether one latent class model is statistically better than another. In addition to the smallest class count and class size in each latent class model, other diagnostic criteria including the lowest value of average latent class posterior probability (ALCPP) and entropy were also adopted to estimate the accuracy of class membership prediction and the classification quality, respectively, across models, where a value close to 1 is ideal. The optimal model was selected based on the model fit indices and the theoretical interpretability of the identified classes, supplemented by the diagnostic criteria. As LCA assumes that class indicators are conditionally independent of each another given class membership, bivariate residual associations (BVR) method based on maximum likelihood estimation was employed to detect assumption violation [25]. Detected residual associations of class indicator pairs with “high” conditional dependence (i.e., a BVR > 10) were then modelled to observe any significant improvement of model fit indices compared with models without residual association modelling [25]. Once the final model was selected, the cardiovascular risk profiles were compared based on the relative proportions of indicators across latent classes. Due to the potentially arbitrary cut-offs for some indicators (e.g., low physical activity and stress), sensitivity analyses were conducted to assess the robustness of the resulted LCA model by adopting alternative threshold values for their binary categorization (Supplementary Figure S1). Multinomial logistic regression was also adopted to identify their differences in sociodemographic characteristics. Listwise deletion was employed for the regression analysis due to small number of missing values in sociodemographic factors (See footnote in Table 1). All statistical analyses (i.e., VLMR LRT and multinomial logistic regression) were two-tailed with a significance level of *p* < 0.05, and conducted using the statistical software Mplus version 8.3 and R version 4.3.1.

## Results

### Descriptive statistics

Among the 1181 sampled South Asian adults, the mean age was 42.7 ± 13.2 years (Table 1). Most participants were female (76.5%) and currently married (86.9%). The average household size was 4.7 ± 1.8 members per household. Almost half were Pakistani (43.9%), followed by Indians (28.8%), Nepalese (21.2%), and others from Bangladesh and Sri Lanka (6.1%). Most participants migrated to Hong Kong for over 7 years (72.4%), in addition to 7.8% participants being born in Hong Kong. About one-third of participants attained no more than primary education (31.6%), whereas around one-fourth attained post-secondary education (26.0%). Also, 62.0% of participants were economically inactive and 16.1% were on CSSA. Regarding lifestyle behaviours, 46.4% and 42.9% of participants did not consume vegetables and fruits, respectively, every day. Slightly over half were less physically active with none or light exercise only (51.9%), whereas 11.0% were either current smoker or ex-smoker. As for psychological symptoms, 36.1% had higher psychological distress and 7.9% showed depressive symptoms. In terms of obesity burden, 54.6% had a BMI ≥ 27.5 kg/m^2^, 90.0% had a WHtR > 0.5, and 66.6% were classified as obese based on body fat percentage. Lastly, the observed prevalence of clinical conditions including hypertension, hypercholesterolemia, and diabetes mellitus were 24.1%, 15.0%, and 12.1%, respectively.

**Table 1:** Basic characteristics of sampled South Asian adults (*N* = 1181)

	N* (Mean)*	%* (SD)*
***Demographic factors***		
Age	*42.7*	*13.2*
Female gender	904	76.3
Currently married	1026	86.9
Household size	*4.7*	*1.8*
Length of stay in Hong Kong^a^		
< *4 years*	108	9.2
* 4–7 years*	124	10.6
> *7 years*	847	72.4
* Born in Hong Kong*	91	7.8
Ethnicity		
* Pakistani*	519	43.9
* Nepalese*	250	21.2
* Indian*	340	28.8
* Others*	72	6.1
***Socioeconomic indicators***		
Education level		
* Primary or below*	373	31.6
* Secondary*	501	42.4
* Post-secondary*	307	26.0
Economically inactive^b^	732	62.0
On CSSA	190	16.1
***Lifestyle behaviours***		
Not eating vegetable every day^c^	547	46.4
Not eating fruits every day	507	42.9
Doing none or light exercise only^d^	611	51.9
Ever-smoker	130	11.0
***Psychological factors***		
Higher psychological distress	422	36.1
Depressive symptoms^e^	93	7.9
***Obesity measures***		
High BMI^f^	640	54.6
High WHtR^g^	1050	90.0
High fat percentage^h^	768	66.6
***Clinical conditions***		
Hypertension^i^	284	24.1
Hypercholesterolemia^j^	175	15.0
Diabetes mellitus^k^	141	12.1

Missing: ^a^ 11; ^b^ 1; ^c^ 2; ^d^ 3; ^e^ 3; ^f^ 9; ^g^ 14; ^h^ 28; ^i^ 5; ^j^ 15; ^k^ 18

### Identification of cardiovascular risk profiles

Model fit indices were compared from 1-class model to 8-class model (Table 2). The 5-class model showed the lowest BIC (14,067.72) and CAIC (14,131.72), whereas the 6-class model had the lowest ssBIC (13,832.91) and the 8-class model had the lowest AIC (13,663.09). As the VLMR likelihood ratio test results suggested no further improvement in models with more than 6 classes, we restricted the model selection between the 5-class and 6-class models. In terms of class distribution and interpretability, the 6-class model generated two relatively small latent classes with only 72 participants (6.1%) each, whereas the smallest class size in the 5-class model reached 105 (8.9%) and no other classes had a size less than 10%. Also, the lowest ALCPP value in the 5-class model was higher than that in the 6-class model (0.816 versus 0.788), indicating a more accurate class membership prediction. Moreover, after modelling the residual associations of the two pairs of class indicators with high BVR (i.e., one pair between BMI and WHtR, and another pair between stress and depressive symptoms), the 5-class residual association model showed similar model fit indices compared with the 6-class model (Supplementary Table S1), indicating that addition of the sixth class may not be necessary. Therefore, although the 6-class model had a slightly higher entropy (0.745 versus 0.735) and statistically significant VLMR likelihood ratio test (*p* = 0.017), the 5-class model with the lowest BIC, deemed as the most reliable model fit statistics, as well as a more balanced and interpretable class distribution, was selected as the optimal classification of cardiovascular risk profiles among the sampled South Asian adults.

**Table 2:** Summary of latent class model identification and model fit indices (*N* = 1181)

	**Model fit indices**						**Diagnostic criteria**				
**Classes**	**Free parameters**	**Log-likelihood**	**AIC**	**BIC**	**CAIC**	**ssBIC**	**Smallest class count**	**Smallest class size (%)**	**Lowest ALCPP by class**	**Entropy**	**VLMR LRT**
1	12	−7430.768	14,885.54	14,946.43	14,958.43	14,908.31	1181	100.0	NA	NA	NA
2	25	−7019.687	14,089.37	14,216.23	14,241.23	14,136.82	356	30.1	0.864	0.889	< .001
3	38	−6932.603	13,941.21	14,134.02	14,172.02	14,013.32	356	30.1	0.841	0.714	< .001
4	51	−6861.214	13,824.43	14,083.21	14,134.21	13,921.21	135	11.4	0.801	0.721	0.0129
5	64	−6807.487	13,742.97	14,067.72	14,131.72	13,864.43	105	8.9	0.816	0.735	0.0722
6	77	−6766.392	13,686.78	14,077.49	14,154.49	13,832.91	72	6.1	0.788	0.745	0.0165
7	90	−6746.019	13,672.04	14,128.71	14,218.71	13,842.84	59	5.0	0.759	0.752	0.5395
8	103	−6728.544	13,663.09	14,185.72	14,288.72	13,858.56	60	5.1	0.716	0.747	0.3332

**Fig. 1:**
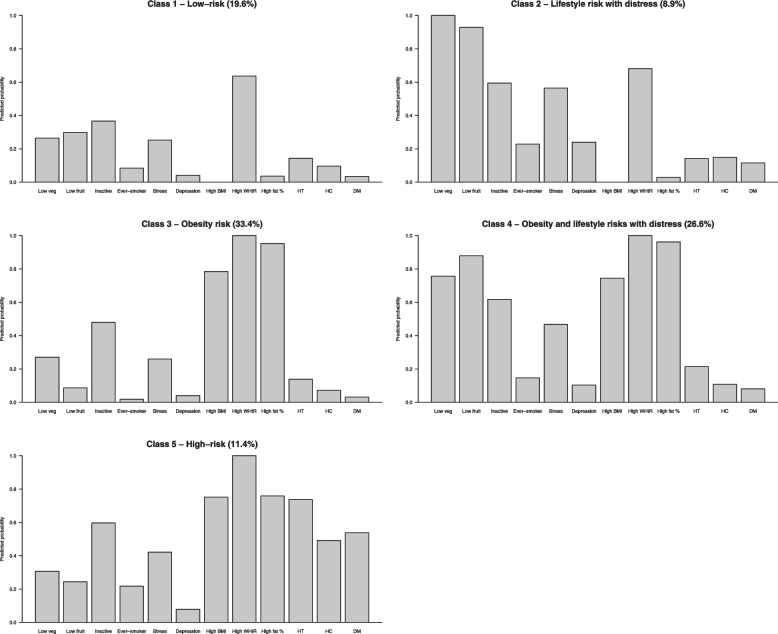
Predicted probabilities of cardiovascular risk profiles by five latent classes. Footnotes: Low veg: Low vegetable intake; Low fruit: Low fruit intake; Inactive: Low physical activity level; BMI: Body mass index; WHtR: Waist-to-height ratio; HT: Hypertension; HC: Hypercholesterolemia; DM: Diabetes mellitus

Latent classes 1–5 were characterized by the different combinations of lifestyle, psychological, obesity, and clinical risks (Fig. 1). Class 1 (19.6%) was labelled as “low-risk” group due to its lowest cardiovascular risk, comprising people with relatively healthy lifestyle behaviours, low psychological symptoms, and low clinical risks except for a moderately high prevalence of abdominal obesity. Class 2 (8.9%) was labelled as “lifestyle risk with distress” group, consisting of people with similar obesity and clinical risk profiles as Class 1 but more psychological symptoms and very high probabilities of having unhealthy lifestyle behaviours. Class 3 (33.4%), labelled as “obesity risk” group, was the largest latent class which had a similarly low-risk profile as Class 1 except for a high obesity burden. Class 4 (26.6%) was labelled as “obesity and lifestyle risks with distress” group, comprising people with more psychological symptoms, very high probabilities of having unhealthy lifestyle behaviours, and obesity but low clinical risks. Lastly, Class 5 (11.4%), labelled as “high-risk” group, was of the highest probabilities of having clinical conditions, obesity, physical inactivity, smoking, and stress. Sensitivity analysis based on the 5-class residual association model showed similar class membership distribution (Supplementary Table S1).

### Sociodemographic characteristics across latent classes

Members of the five latent classes significantly differed in terms of demographic, ethnic, and socioeconomic profiles (Table 3). First, members in “high-risk” Class 5 were significantly older than those in “low-risk” Class 1 (aOR = 1.11 [95% CI = 1.05, 1.16]), despite no significant age differences among the other classes. Second, with reference to “low-risk” Class 1, female gender was positively associated with “obesity risk” Class 3 (aOR = 2.72 [1.27, 5.81]), but negatively with “lifestyle risk with distress” Class 2 (aOR = 0.33 [0.14, 0.78]) and “high-risk” Class 5 (aOR = 0.22 [0.07, 0.74]) compared to male gender. Third, apparent ethnic differences were observed among classes. Relative to Indians, Pakistani and/or Nepalese tended to be members in higher risk classes including “lifestyle risk with distress” Class 2 (Pakistani: aOR = 8.03 [2.66, 24.28]), “obesity risk” Class 3 (Pakistani: aOR = 3.12 [1.62, 6.04]; Nepalese: aOR = 2.18 [1.20, 3.99]), “obesity and lifestyle risks with distress” Class 4 (Pakistani: aOR = 3.65 [1.89, 7.06]; Nepalese: aOR = 2.22 [1.23, 4.00]), and “high-risk” Class 5 (Nepalese: aOR = 2.73 [1.08, 6.94]). Regarding the socioeconomic differences, there is an indication of lower education level in higher risk classes. Specifically, members in “high-risk” Class 5 had significantly lower education level than those in “low-risk” Class 1 (secondary: aOR = 0.11 [0.04, 0.31]; post-secondary: aOR = 0.20 [0.05, 0.79]). In addition, members in “obesity risk” Class 3 tended to be married (aOR = 2.42 [1.06, 5.51]), and born outside Hong Kong (migrated to Hong Kong for < 4 years: aOR = 2.78 [1.05, 7.34]; migrated to Hong Kong for > 7 years: aOR = 2.59 [1.20, 5.59], with reference to those born in Hong Kong) when compared to “low-risk” Class 1. It is also worth noting that members in “obesity risk” Class 3 tended to be economically inactive (aOR = 1.83 [1.07, 3.14]) compared to both “low-risk” Class 1 and “obesity and lifestyle risks with distress” Class 4 characterized by having more unhealthy lifestyle behaviours and distress in addition to obesity (aOR = 2.33 [1.38, 3.92]; data not shown in Table 3). Sensitivity analysis based on the 5-class residual association model showed highly consistent findings (Supplementary Table S2).

**Table 3:** Associations of demographic and socioeconomic characteristics with latent class membership with reference to low-risk Class 1 based on multinomial logistic regression

	**Class 2**	**Class 3**	**Class 4**	**Class 5**
	(Lifestyle risk with distress)	(Obesity risk)	(Obesity and Lifestyle risks with distress)	(High-risk)
	aOR (95% CI)	aOR (95% CI)	aOR (95% CI)	aOR (95% CI)
Age	0.99 (0.93, 1.06)	1.00 (0.97, 1.03)	1.01 (0.98, 1.04)	1.11 (1.05, 1.16)
Female gender	0.33 (0.14, 0.78)	2.72 (1.27, 5.81)	0.96 (0.52, 1.79)	0.22 (0.07, 0.74)
Currently married	0.59 (0.20, 1.73)	2.42 (1.06, 5.51)	1.26 (0.63, 2.50)	0.63 (0.16, 2.45)
Household size	1.04 (0.85, 1.26)	1.11 (0.98, 1.27)	1.09 (0.95, 1.26)	1.06 (0.86, 1.31)
Ethnicity				
* Indian*	ref	ref	ref	ref
* Pakistani*	8.03 (2.66, 24.28)	3.12 (1.62, 6.04)	3.65 (1.89, 7.06)	1.76 (0.53, 5.88)
* Nepalese*	2.79 (0.82, 9.50)	2.18 (1.20, 3.99)	2.22 (1.23, 4.00)	2.73 (1.08, 6.94)
* Others*	1.44 (0.27, 7.64)	1.73 (0.73, 4.07)	1.00 (0.40, 2.52)	1.15 (0.29, 4.55)
Length of stay in Hong Kong				
Born in Hong Kong	ref	ref	ref	ref
< 4 years	1.15 (0.24, 5.44)	2.78 (1.05, 7.34)	0.74 (0.27, 1.98)	0.52 (0.06, 4.56)
4–7 years	3.23 (0.82, 12.69)	2.67 (0.99, 7.21)	1.70 (0.68, 4.28)	1.30 (0.22, 7.77)
> 7 years	0.98 (0.28, 3.41)	2.59 (1.20, 5.59)	1.29 (0.64, 2.61)	1.61 (0.39, 6.72)
Education				
* Primary or below*	ref	ref	ref	ref
* Secondary*	0.90 (0.32, 2.54)	0.76 (0.42, 1.39)	0.56 (0.31, 1.00)	0.11 (0.04, 0.31)
* Post-secondary*	1.94 (0.61, 6.14)	1.00 (0.48, 2.08)	1.04 (0.52, 2.07)	0.20 (0.05, 0.79)
Economically inactive	0.99 (0.38, 2.57)	1.83 (1.07, 3.14)	0.79 (0.45, 1.38)	1.34 (0.49, 3.66)
On CSSA	0.66 (0.17, 2.50)	1.29 (0.64, 2.63)	1.57 (0.76, 3.23)	1.69 (0.58, 5.00)

## Discussion

As the first study to apply an intersectional approach to assess the cardiovascular risk profiles among the South Asian community in Hong Kong, we identified five latent classes with distinct combinations of cardiovascular risk factors and sociodemographic features. Apart from the higher clinical risk in the older adults, women tended to be clustered into classes with high obesity burden while men, and also the economically active, were more likely to be grouped with poorer lifestyles in general. Ethnic differences were also obvious within the South Asian community; Pakistani and Nepalese were consistently associated with being in the higher risk classes compared to Indians. In addition, educational attainment and economic activity status were the socioeconomic indicators that showed associations with cardiovascular risk profiles among our South Asian participants.

Our findings on the differential risk factors and sociodemographic profiles across latent classes provide several noteworthy insights into health promotion strategies for the South Asian community in Hong Kong. First, the common observation that psychological symptoms tend to cluster with unhealthy lifestyle and clinical conditions [26] appears to hold true among our South Asian participants (see Class 2 and Class 4 in Fig. 1). Apart from the prevalent co-morbid mental health disorders in patients with cardiovascular conditions [27], our findings echoed previous studies supporting that lifestyle behaviours may change as a function of stress. For example, a recent meta-analysis concluded a modest yet evident association of stress with intake of more unhealthy foods but less healthy foods in apparently healthy adults [26], whereas another earlier review study showed that experience of stress impairs individual’s efforts to engage in sports and exercise just as it negatively affects smoking and other risky health behaviours [28]. Applying such findings into community practice, public health practitioners could pay extra attention to mental health issues for the South Asian community who are screened with unhealthy lifestyle [29]. Incorporating stress management into lifestyle interventions may alleviate stress-related unhealthy behaviours.

Second, significant heterogeneity in cardiovascular risk profiling exists within our South Asian participants. As shown in our results, Pakistani and Nepalese tended to be clustered into higher risk classes compared to Indians after adjustment for age and socioeconomic differences among others. Our findings are largely consistent with previous research supporting a poorer cardiovascular risk profiles in terms of obesity and physical activity among Pakistani than Indians living in developed Western settings [30–32]. Also, based on the UK Biobank prospective cohort study, Indians appeared to have relatively lower atherosclerotic cardiovascular risk among the South Asian ethnic groups, despite having a higher risk compared to individuals of European ancestry [33]. These ethnic differences in cardiovascular risks within South Asians reiterated the need to dismiss the prevailing stereotype and community perception that assumes all South Asians are a homogeneous group for health promotion. Their subtle differences in cultural values, social norm, behavioural routines, and health risks [34, 35] should be considered when developing culturally tailored programmes for different South Asian ethnic groups, in particular Pakistani and Nepalese in Hong Kong with heightened cardiovascular risks.

Third, lifestyle risks appear to be higher among South Asian men than their female counterparts in our sample. Notably, when compared to Class 3 (i.e., obesity risk only), members of Class 4, characterized by both obesity and lifestyle risks, tended to be male and economically active. Such result is concerning because South Asian men, especially those employed, are often the most difficult to reach in the community. The full potential of any lifestyle interventions could hardly be realized unless these specific subgroups of South Asians are included. Meanwhile, over-emphasis on lifestyle changes among economically inactive women may also overlook other possible cultural reasons behind their obesity burden such as gender roles, social norm on a larger body size [36], as well as the potential weight retention associated with high parity among South Asian women [37, 38]. Despite possible selection biases in our sample, the observed potential gender difference in lifestyle patterns deserves more careful considerations in designing recruitment and health promotion strategies to better cater for the needs of South Asian men.

Fourth, among the socioeconomic indicators, educational attainment appeared to be associated with risk profiling; the higher the cardiovascular risks of a latent class, the less likely to observe South Asian participants with secondary education or above. Such finding is in line with the well-established evidence on the strong and consistent relationship of education with cardiovascular health and its risk factors [39]. Educational attainment does not only determine access to economic resources since young adulthood, but also has numerous health implications on one’s health literacy to make informed decisions, cognitive skills and ability to benefit from new health knowledge, problem-solving capability, care-seeking behaviours, and income for investment on healthy food and lifestyle, which are all conducive to cardiovascular health [40, 41]. Culturally sensitive empowerment programmes targeting South Asians who are less educated may be promising to address their health and social needs coupled with socioeconomic disadvantages.

Last but not least, it is worth noting that over half of our South Asian participants had none or light exercise only, and the relative difference in low physical activity across classes (ranging from 36.6% to 61.8%) was not as apparent as in other risk factors. Even in “low-risk” class 1, more than one-third did not have any moderate or vigorous exercises, suggesting that improving physical activity may be a universal target for health promotion regardless of risk profiles. Despite no local studies for comparison, earlier research in the United Kingdom supported the very low physical activity level among South Asians compared to the white, independent of their socioeconomic differences [31]. The authors speculated that in addition to urbanization and psychological distress, cultural barriers may also partially explain their particularly low physical activity level. For example, language barriers, preference on larger body size, and religious belief that health and death is beyond one’s own control but determined by the God, were identified in earlier studies [30, 36]. Specifically for South Asian women, additional barriers include time constraints due to familial duties, culturally inappropriate facilities with gender concerns, and stigma arising from others in their community that taking time for exercise, rather than caretaking, is a selfish act [30].The cultural explanation may be particularly relevant given that second-generation South Asians, who are generally more adapted to the host culture, tend to engage more in physical activity than the first-generation migrants [42]. These findings highlighted the criticality of enhancing cultural sensitivity and fostering engagement with community stakeholders (e.g., South Asian members, frontline workers, religious leaders) as a co-creation process in developing health programmes for promoting physical activity among the South Asian community.

## Limitations

The study has several caveats. First, the lack of a random sampling frame may limit the representativeness of the South Asian community in Hong Kong and the generalizability of our results. For example, this study recruited proportionally less Indian participants (28.8% in this study versus 41.7% in Hong Kong) but more Pakistani participants (43.9% versus 23.9%) compared to the Population Census on ethnic minorities in Hong Kong [8]. Also, as the sample was dominated by female participants, further studies targeting more South Asian men are warranted to verify the gender-related observations across latent classes. Second, the list of cardiovascular risk factors included in this study may not be exhaustive due to data availability. The use of binary thresholds may also be arbitrary for certain risk conditions even though the robustness of the LCA model was confirmed through sensitivity analyses. Third, the reliance on self-reported data to assess lifestyle behaviours and psychological symptoms may possibly introduce recall and social desirability biases. Last, due to the cross-sectional nature, causal relationships cannot be inferred from the findings.

## Conclusion

This study extended the existing literature on cardiovascular risk profiling among the South Asian community in Hong Kong and added new insights into the distinct risk patterns and sociodemographic features across the five identified latent classes. Although no causal inferences could be established in this study, our findings suggested several priority areas for actions and important implications into the strategies for targeted community programmes on cardiovascular health of this socially vulnerable population. While the South Asian community in Hong Kong is still relatively young, our observed heavy burden on obesity, lifestyle risks especially in men, and low overall physical activity level could possibly be translated into a tremendous cardiovascular disease burden in the forthcoming decades, in particular among Pakistani and Nepalese. Prompt interventions or policy actions, with adequate community engagement and cultural sensitivity, are therefore warranted to cater for the unique needs of different groups of people within the South Asian community in Hong Kong.

## Supplementary Information

Supplementary Material 1.

## Data Availability

The datasets used and/or analysed during the current study are available from the corresponding author on reasonable request.
