# Supplementary material for: Cardiovascular risk profiling among South Asian adults in Hong Kong: a latent class analysis
Source: Int J Equity Health. 2025 Jan 17;24:14. doi: 10.1186/s12939-025-02376-8 (PMC11740419; doi:10.1186/s12939-025-02376-8)
Supplement: Supplementary file 1 — Supplementary Material 1. [file 12939_2025_2376_MOESM1_ESM.docx]

**Sensitivity analyses on the latent class analyses**

To assess the robustness of the 5-class model presented in the main text, we have conducted sensitivity analyses by separately changing the arbitrary cut-off points for low physical activity level (from no moderate or vigorous exercise to no vigorous exercise) and stress (from a cut-off at 4 to 3 out of the 6-point Likert scale). Results showed highly similar proportions of the 5 latent classes and their corresponding 12 risk indicators in these two amended models compared to the original model (see the figure below for the consistent patterns), indicating the robustness of classification and interpretation of our 5-class model.

Figure S1. Comparison between the original 5-class model with two amended models


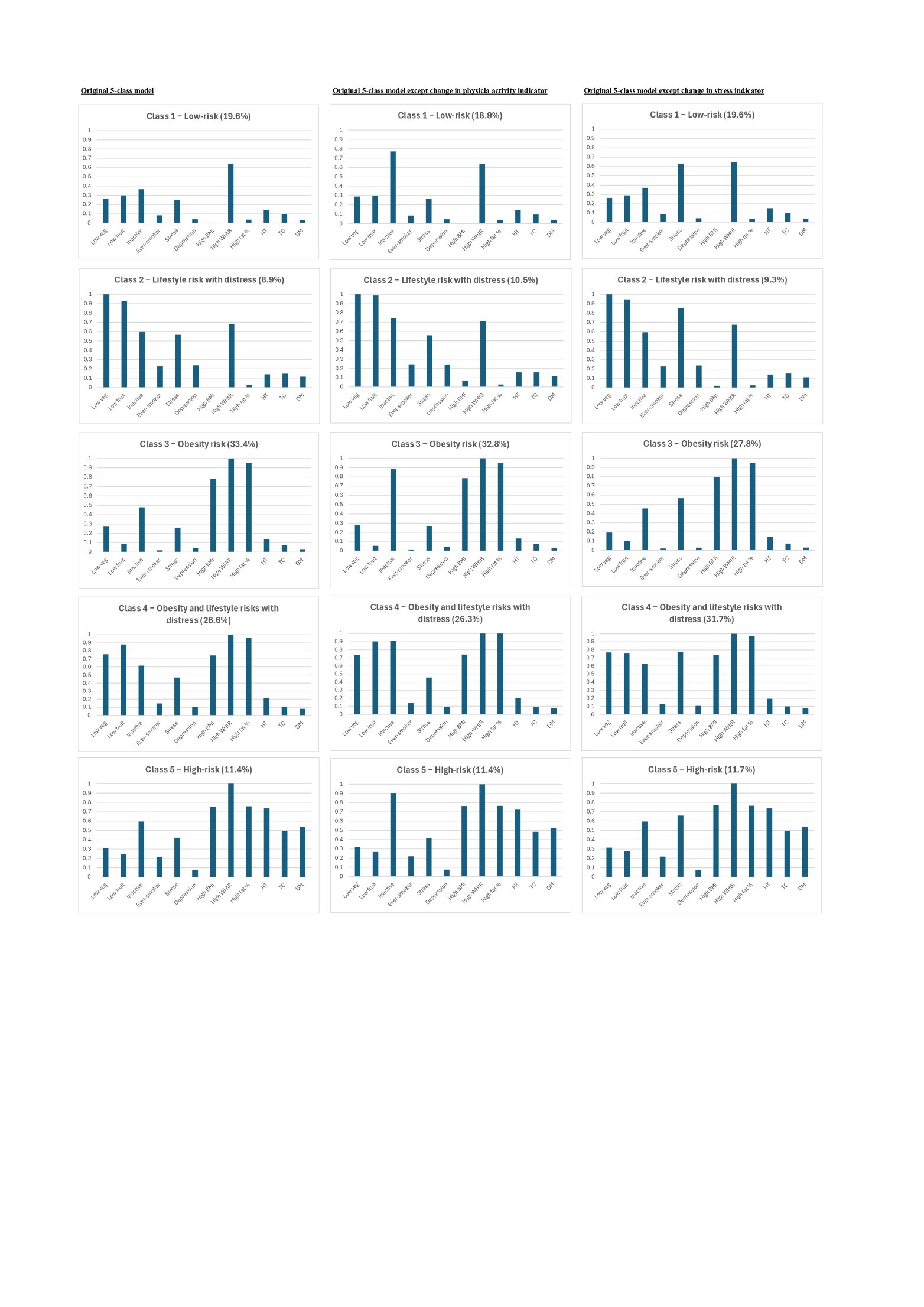


**Sensitivity analyses by considering conditional dependence using residual association model**

As LCA assumes that class indicators are conditionally independent of each another given class membership, we followed the detection and modelling methods proposed by Visser and Depaoli (2022) and re-run our LCA model for sensitivity analysis. To detect conditional dependence across class indicators, the bivariate residual associations (BVR) method based on maximum likelihood estimation was employed in MPlus. Since there is no rule of thumb to indicate “high” BVR, we adopted a threshold value >10 used in Visser and Depaoli (2022), which gives stronger confidence on detecting non-zero residual correlations and offers greatest improvement to model fit when later modelling the detected residual associations with BVR >10. After re-running the 5-class model, we found two pairs of class indicators with their BVR >10 (i.e., one pair between BMI and WHtR, and another pair between stress and depressive symptoms).

As failure to take conditional dependence into account statistically may lead to biased parameter estimates and unreliable model fit indices, we conducted a sensitivity analysis by modelling conditional dependence using the 5-class LCA residual association model based on maximum likelihood estimation. As shown in Table S1, while the model fit indices in the 5-class LCA residual association model were slightly better than those in the 5-class classical model, its class membership distribution remained highly similar to the 5-class classical model. Comparing the 6-class classical model with the two 5-class models, the model fit indices were more or less similar that the 5-class models showed better BIC while the 6-class model showed better AIC. Their similar model fit indices indicated no apparent improvement in model fit if selecting the 6-class model over the 5-class model. However, the 6-class model generated two small classes with N<100, which may hamper the interpretability of class memberships. Given the above, our decision on choosing the 5-class model over the 6-class model holds valid, even after taking conditional dependence into account.

| **Table S1. Model fit indices and class prevalence for 5-class classical model, 5-class residual association model, and 6-class classical model** | | | | | | |
| --- | --- | --- | --- | --- | --- | --- |
|  |  | 5-class classical LCA model |  | 5-class LCA residual association model |  | 6-class classical LCA model |
| ***Model fit*** | |  |  |  |  |  |
|  | Log-likelihood | -6807.487 |  | -6795.978 |  | -6766.392 |
|  | AIC | 13742.974 |  | 13723.956 |  | 13686.784 |
|  | BIC | 14067.71748 |  | 14058.848 |  | 14077.491 |
|  | ssBIC | 13864.431 |  | 13849.208 |  | 13832.911 |
|  | Entropy | 0.735 |  | 0.753 |  | 0.745 |
| ***Class prevalence (N; %)*** | |  |  |  |  |  |
|  | Class 1 | 232; 19.6% |  | 234; 19.8% |  | 230; 19.5% |
|  | Class 2 | 105; 8.9% |  | 111; 9.4% |  | 72; 6.1% |
|  | Class 3 | 395; 33.4% |  | 408; 34.5% |  | 381; 32.3% |
|  | Class 4 | 314; 26.6% |  | 295; 25.0% |  | 291; 24.6% |
|  | Class 5 | 135; 11.4% |  | 133; 11.3% |  | 135; 11.4% |
|  | Class 6 | NA |  | NA |  | 72; 6.1% |

We have also re-run the multinomial logistic regression across the 5 classes generated by the residual association model (see Table S2), and found highly consistent pattern of demographic and socioeconomic predictors of class membership compared with the original result as shown in Table 3. Such consistency reassured the rigour of our 5-class LCA model.

| **Table S2. Associations of demographic and socioeconomic characteristics with latent class membership with reference to low-risk Class 1 based on multinomial logistic regression** | | | | | | | | | |
| --- | --- | --- | --- | --- | --- | --- | --- | --- | --- |
|  |  |  | **Class 2** |  | **Class 3** |  | **Class 4** |  | **Class 5** |
|  |  |  | (Lifestyle risk with distress) |  | (Obesity risk) |  | (Obesity and Lifestyle  risks with distress) |  | (High-risk) |
|  |  |  | aOR (95% CI) |  | aOR (95% CI) |  | aOR (95% CI) |  | aOR (95% CI) |
| Age | |  | 1.00 (0.94, 1.06) |  | 1.00 (0.97, 1.02) |  | 1.01 (0.98, 1.04) |  | 1.11 (1.05, 1.16) |
| Female gender | |  | 0.33 (0.15, 0.77) |  | 2.53 (1.29, 4.95) |  | 0.92 (0.49, 1.73) |  | 0.22 (0.07, 0.71) |
| Currently married | |  | 0.61 (0.22, 1.72) |  | 2.45 (1.13, 5.31) |  | 1.18 (0.58, 2.36) |  | 0.56 (0.15, 2.10) |
| Household size | |  | 1.05 (0.86, 1.28) |  | 1.12 (0.98, 1.27) |  | 1.10 (0.96, 1.27) |  | 1.07 (0.86, 1.32) |
| Ethnicity | |  |  |  |  |  |  |  |  |
|  | *Indian* |  | ref |  | ref |  | ref |  | ref |
|  | *Pakistani* |  | 6.18 (2.16, 17.74) |  | 2.65 (1.42, 4.94) |  | 4.08 (2.07, 8.06) |  | 1.69 (0.51, 5.67) |
|  | *Nepalese* |  | 2.67 (0.88, 8.13) |  | 1.89 (1.07, 3.33) |  | 2.60 (1.39, 4.85) |  | 2.86 (1.14, 7.21) |
|  | *Others* |  | 1.89 (0.44, 8.21) |  | 1.44 (0.63, 3.27) |  | 1.04 (0.37, 2.89) |  | 1.19 (0.31, 4.53) |
| Length of stay in Hong Kong | |  |  |  |  |  |  |  |  |
|  | Born in Hong Kong |  | ref |  | ref |  | ref |  | ref |
|  | < 4 years |  | 0.78 (0.17, 3.60) |  | 2.69 (1.06, 6.84) |  | 0.69 (0.24, 1.98) |  | 0.53 (0.07, 4.37) |
|  | 4-7 years |  | 2.24 (0.62, 8.13) |  | 2.70 (1.04, 6.96) |  | 1.75 (0.68, 4.50) |  | 1.19 (0.21, 6.65) |
|  | > 7 years |  | 0.70 (0.22, 2.27) |  | 2.56 (1.23, 5.34) |  | 1.32 (0.65, 2.71) |  | 1.55 (0.40, 5.94) |
| Education | |  |  |  |  |  |  |  |  |
|  | *Primary or below* |  | ref |  | ref |  | ref |  | ref |
|  | *Secondary* |  | 0.86 (0.32, 2.31) |  | 0.72 (0.40, 1.29) |  | 0.53 (0.30, 0.95) |  | 0.12 (0.04, 0.32) |
|  | *Post-secondary* |  | 1.83 (0.60, 5.57) |  | 0.96 (0.47, 1.94) |  | 0.94 (0.47, 1.88) |  | 0.21 (0.06, 0.80) |
| Economically inactive | |  | 0.99 (0.41, 2.37) |  | 1.66 (0.99, 2.77) |  | 0.84 (0.47, 1.49) |  | 1.33 (0.49, 3.63) |
| On CSSA | |  | 0.61 (0.16, 2.38) |  | 1.30 (0.67, 2.56) |  | 1.38 (0.67, 2.86) |  | 1.57 (0.55, 4.55) |

**Reference:**

Visser, M., & Depaoli, S. (2022). A Guide to Detecting and Modeling Local Dependence in Latent Class Analysis Models. Structural Equation Modeling: A Multidisciplinary Journal, 29(6), 971–982.
